# Supplementary material for: Single-cell analysis reveals that stochasticity and paracrine signaling control interferon-alpha production by plasmacytoid dendritic cells
Source: Nat Commun. 2018 Aug 20;9:3317. doi: 10.1038/s41467-018-05784-3 (PMC6102223; doi:10.1038/s41467-018-05784-3)
Supplement: Supplementary file 1 — Supplementary Information [file 41467_2018_5784_MOESM1_ESM.pdf]

Supplementary Information

Single-cell analysis reveals that stochasticity and paracrine signaling control interferon-alpha production by plasmacytoid dendritic cells

Florian Wimmers, Nikita Subedi, Nicole van Buuringen, Daan Heister, Judith Vivié, Inge Beeren-Reinieren, Rob Woestenenk, Harry Dolstra, Aigars Piruska, Joannes F.M. Jacobs, Alexander van Oudenaarden, Carl G. Figdor*, Wilhelm T.S. Huck*, I. Jolanda M. de Vries*, Jurjen Tel#

* Authors contributed equally

# Corresponding author

Supplementary Figures

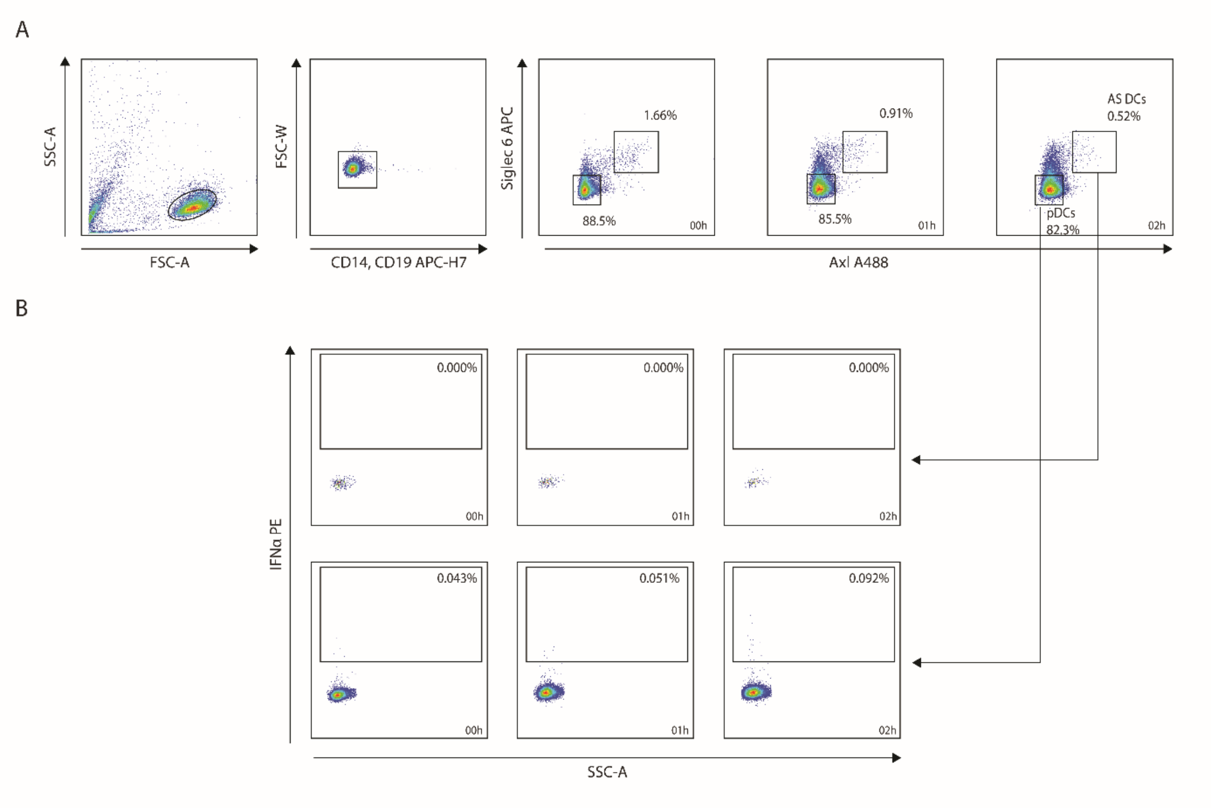

Supplementary Figure 1 – Axl expression by pDCs vanishes early after stimulation with CpG-C and AS DCs produce no IFNα. PDCs were coated with capture reagent, encapsulated in picoliter droplets, and stimulated individually with 50 µg/mL CpG-C. A) After staining for viability, surface marker expression and cytokine secretion, AS DCs were detected via flow cytometry. B) Cytokine expression in AS DCs and traditional pDCs was analyzed.

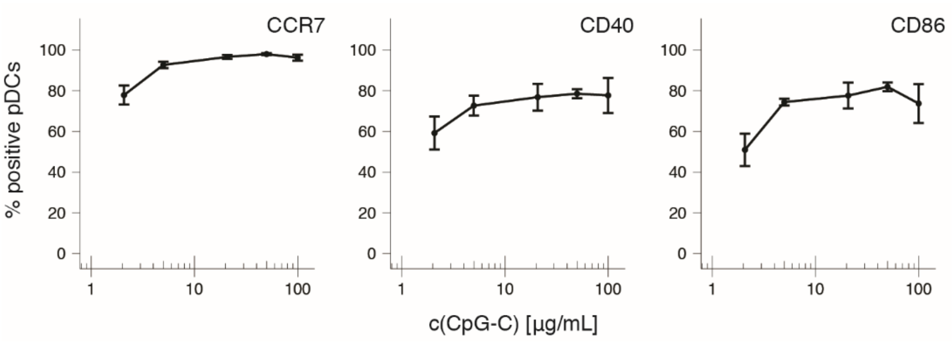

Supplementary Figure 2 – Expression of cell surface markers by individually stimulated pDCs is depending on CpG-C concentration. PDCs were coated with capture reagent, encapsulated in picoliter droplets, and stimulated individually with CpG-C for 12h. After staining for viability, surface marker expression and cytokine secretion, CCR7-, CD40- and CD86-expressing cells were detected via flow cytometry. Shown is the fraction of surface marker-expressing cells plotted against CpG-C concentration. Different concentrations were tested in different donors. Dots indicate mean, error bars indicate SEM. n>=3

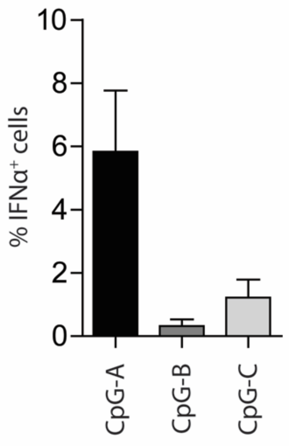

Supplementary Figure 3 – IFNα expression by pDCs stimulated with different CpG molecules. PDCs were coated with capture reagent, encapsulated in picoliter droplets, and stimulated individually with 50 µg/mL CpG-A, -B or -C for 12h. After staining for viability and cytokine secretion, IFNα-secreting cells were detected via flow cytometry. Shown is the fraction of IFNα-secreting cells plotted against treatment condition. Bars indicate mean, error bars indicate SEM. n=2

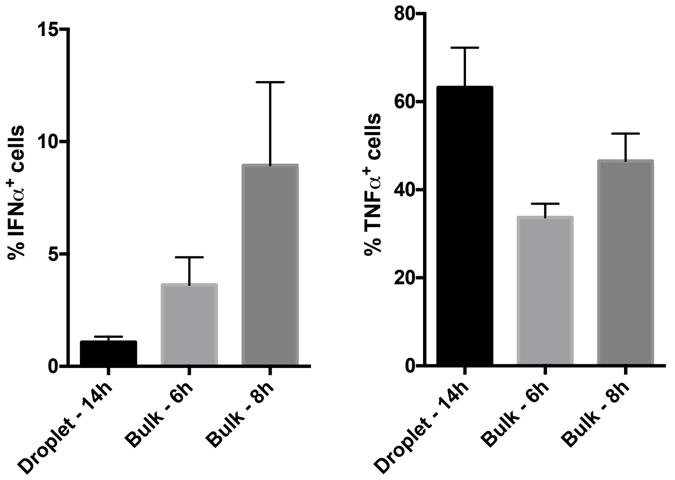

Supplementary Figure 4 – IFNα and TNFα expression by single or bulk activated pDCs from the same donor. PDCs were encapsulated in picoliter droplets and stimulated individually with 50 µg/mL CpG-C for 14h (black bars). Alternatively, pDCs were stimulated with CpG-C in microtiter plates at a density of 25.000 cells/well. After incubation, cells were fixed, permeabilized, and stained for viability and cytokine expression. IFNα- and TNFα-expressing cells were detected using flow cytometry. Shown is the fraction of cytokine-expressing pDCs after 6 hours (light grey bars) or 8 hours (dark grey bars) of incubation. Bars indicate mean, error bars indicate SEM. n=6

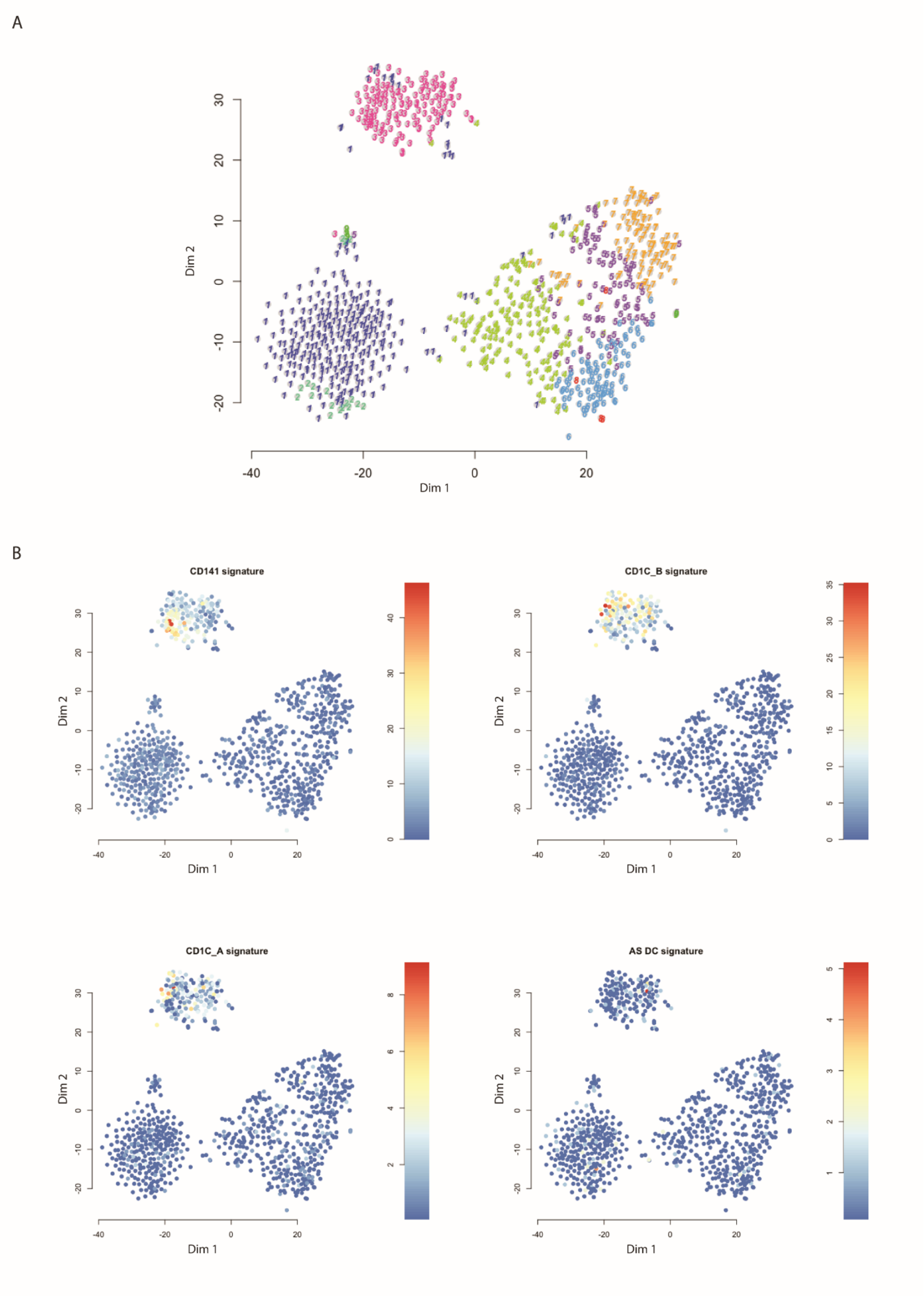

Supplementary Figure 5 – A subset of cells analyzed by scRNA-seq expressed gene signatures from DC subsets other than pDCs. A) t-SNE map showing different DC clusters after initial quality control

filtering, cell clustering using k-medoids, and raceID2 (n = 915, see Methods). B) Expression of different gene signatures by each analyzed cell. Gene signatures are derived from Villani et al.[1] CD141: CLEC9A, HLA-DPA1, CADM1, CAMK2D; CD1C_B: S100A9, S100A8, VCAN, LYZ, ANXA1; CD1C_A: CD1C, FCER1A, CLEC10A, ADAM8, CD1D; AS DC: AXL, SIGLEC6, PPP1R14A, CD22, DAB2.

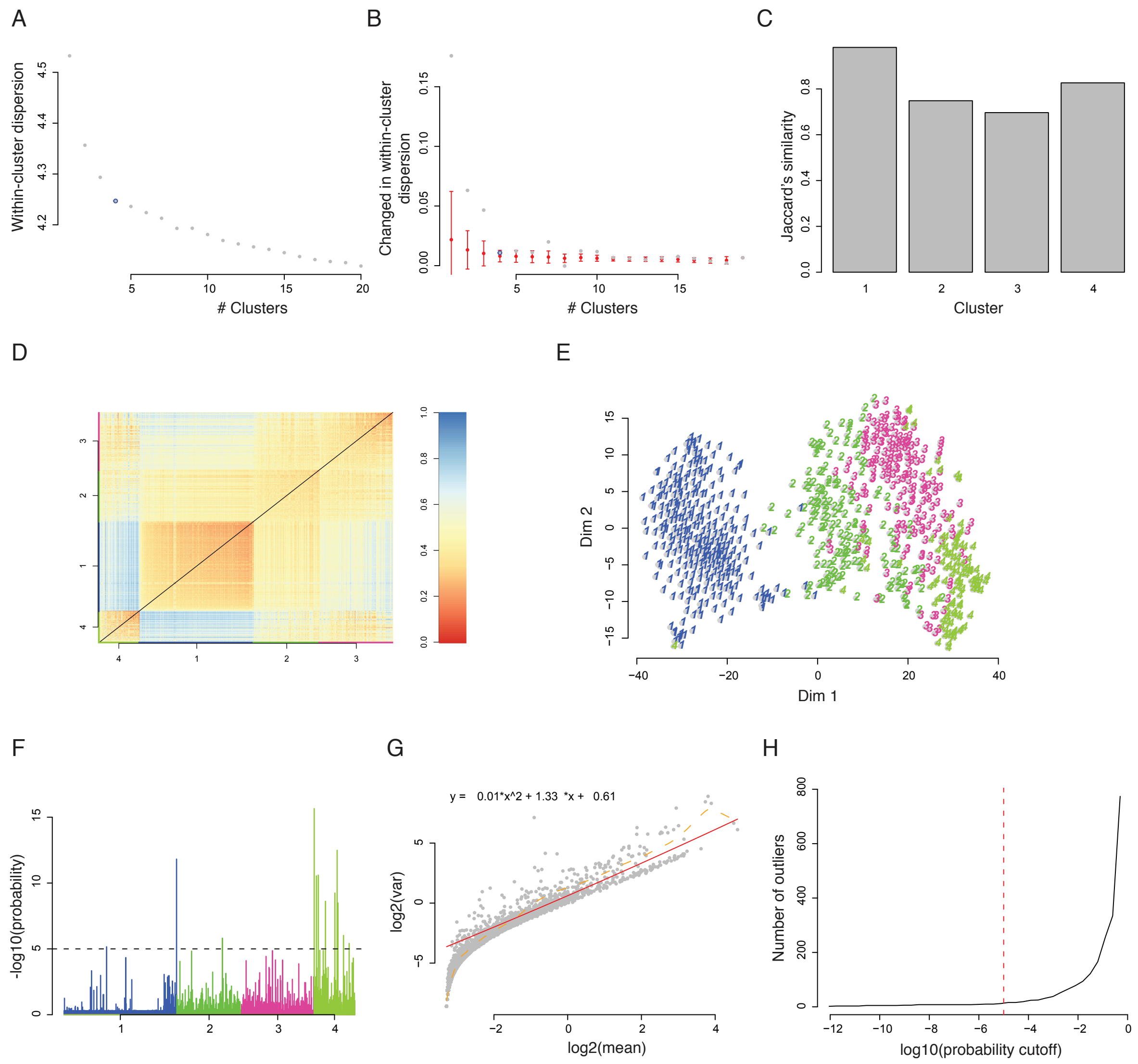

Supplementary Figure 6 – K-medoids clustering and raceID2 of unstimulated and early stimulated pDCs. A, B) Shown is the simulated within-cluster dispersion (A) or its change (B) for a range of seed cluster numbers in k-medoids clustering. N(bootstrapping) = 50. C) The bars indicate the Jaccard's similarity for each cluster identified by k-medoids clustering. D) Heat map of the 774 cells that passed quality control filters representing transcriptome similarities as measured by Euclidean distance. K-medoids clustering characterized 4 clusters based on input from A) - C). E) t-SNE map of different clusters. F) Histogram showing the –log10 probability that transcript levels in a particular cell are explained by a background model (G) accounting for the expected variability. The probability threshold for outlier identification (10^{-5}) is included (black broken line). G) Background model for expected variability. Shown is the log2 variance plotted against the log2 mean. H) The number of the potential outlier cells plotted against the log10 probability threshold is indicated.

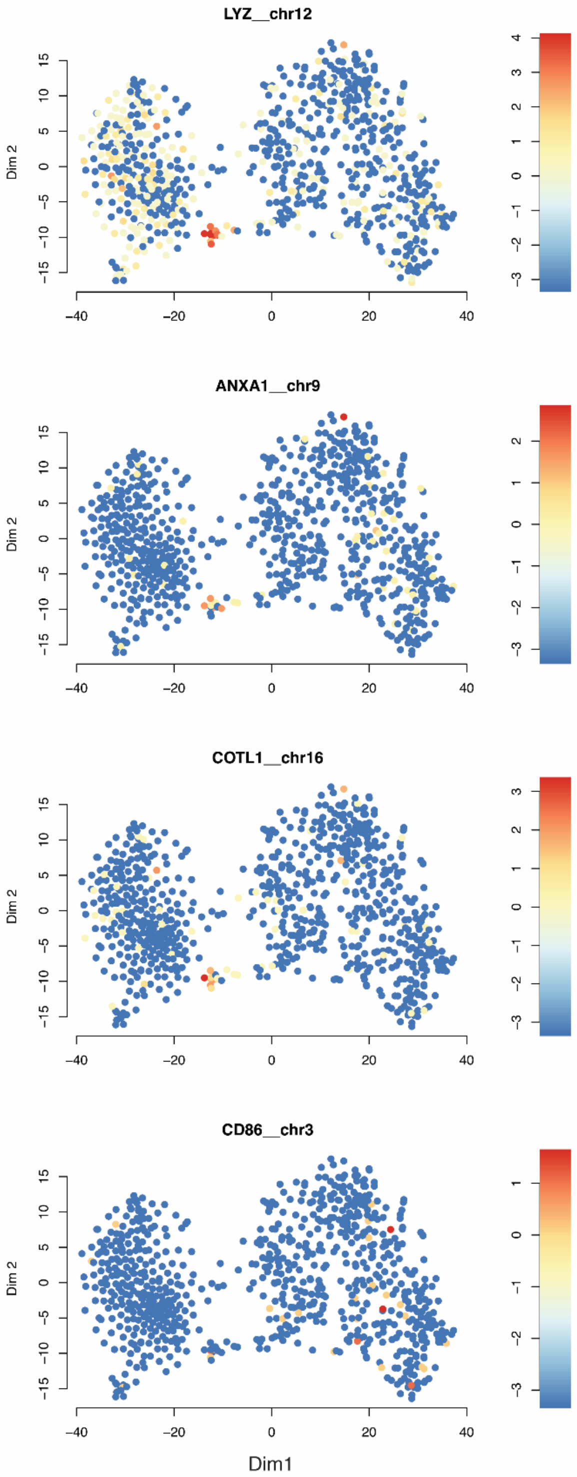

Supplementary Figure 7 – A subset of cells analyzed by scRNA-seq expressed gene signatures of the CD2^{hi} pDC subset. t-SNE map showing different DC clusters after quality control filtering, cell clustering using k-medoids, and raceID2 (see Methods). Color scale indicates the expression values of several genes associated with the CD2^{hi} pDC subset.

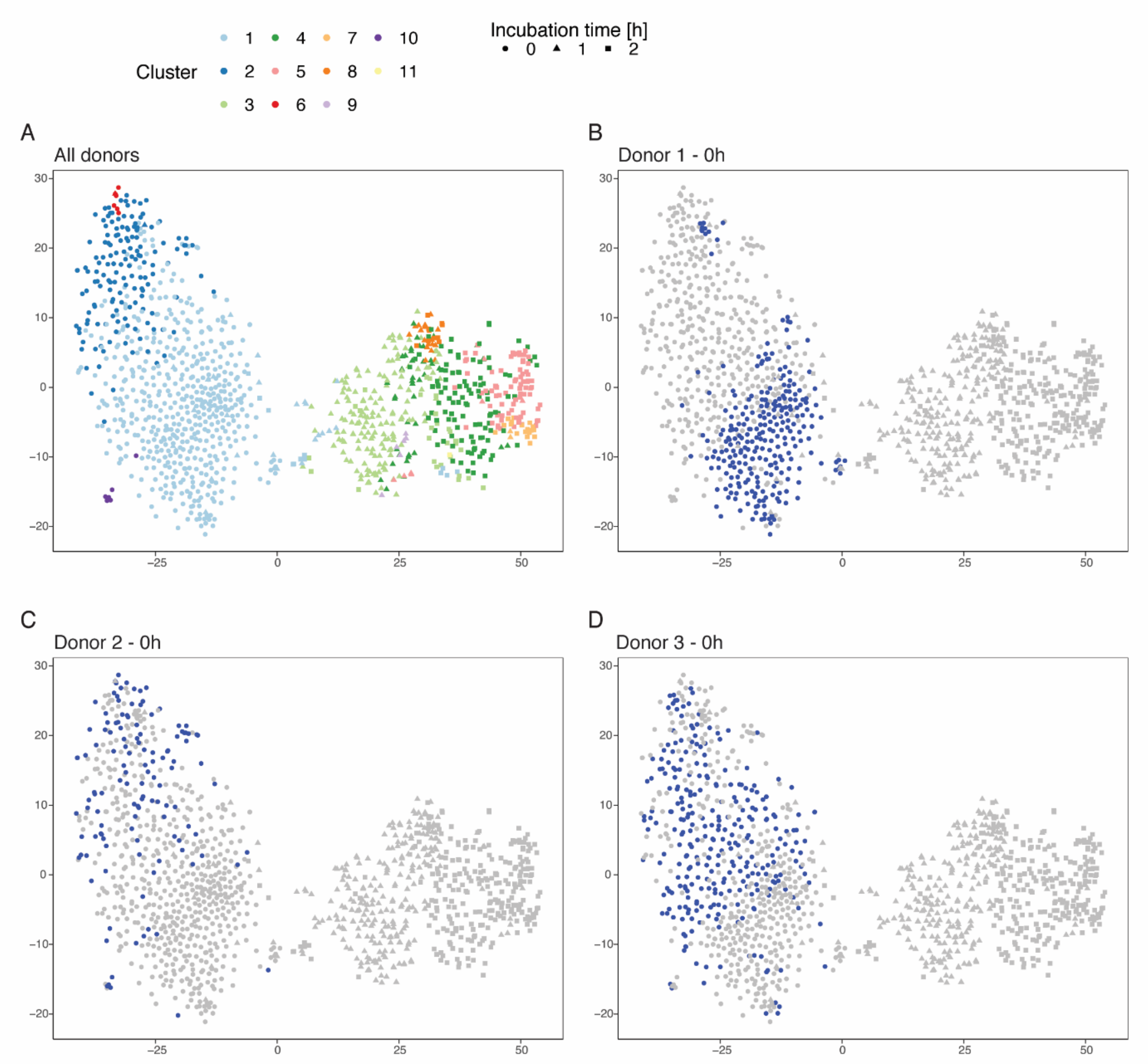

Supplementary Figure 8 – Single cell RNA-sequencing of pDCs from additional healthy donors. PDCs from two additional donors were isolated from PBMCs, collected using fluorescence activated cell-sorting (FACS), and their transcriptomes were sequenced using the SORT-Seq protocol. Single cell transcripts were pooled with Figure 3, and all cells were analyzed together using the previously established filtering pipeline. In total, 1,190 cells expressing 14,979 genes were then subjected to k-medoids clustering and raceID2 analysis using the previously established clustering parameters. A) t-SNE map with identified clusters. Different colors indicate clusters, different shapes indicate stimulation time. Cells in Cl7 show a differential gene expression profile that is similar to cells in Cl5 in Figure 3 (data not shown). B-D) Same t-SNE map as in A). Blue color indicates location of unstimulated pDCs from a particular donor.

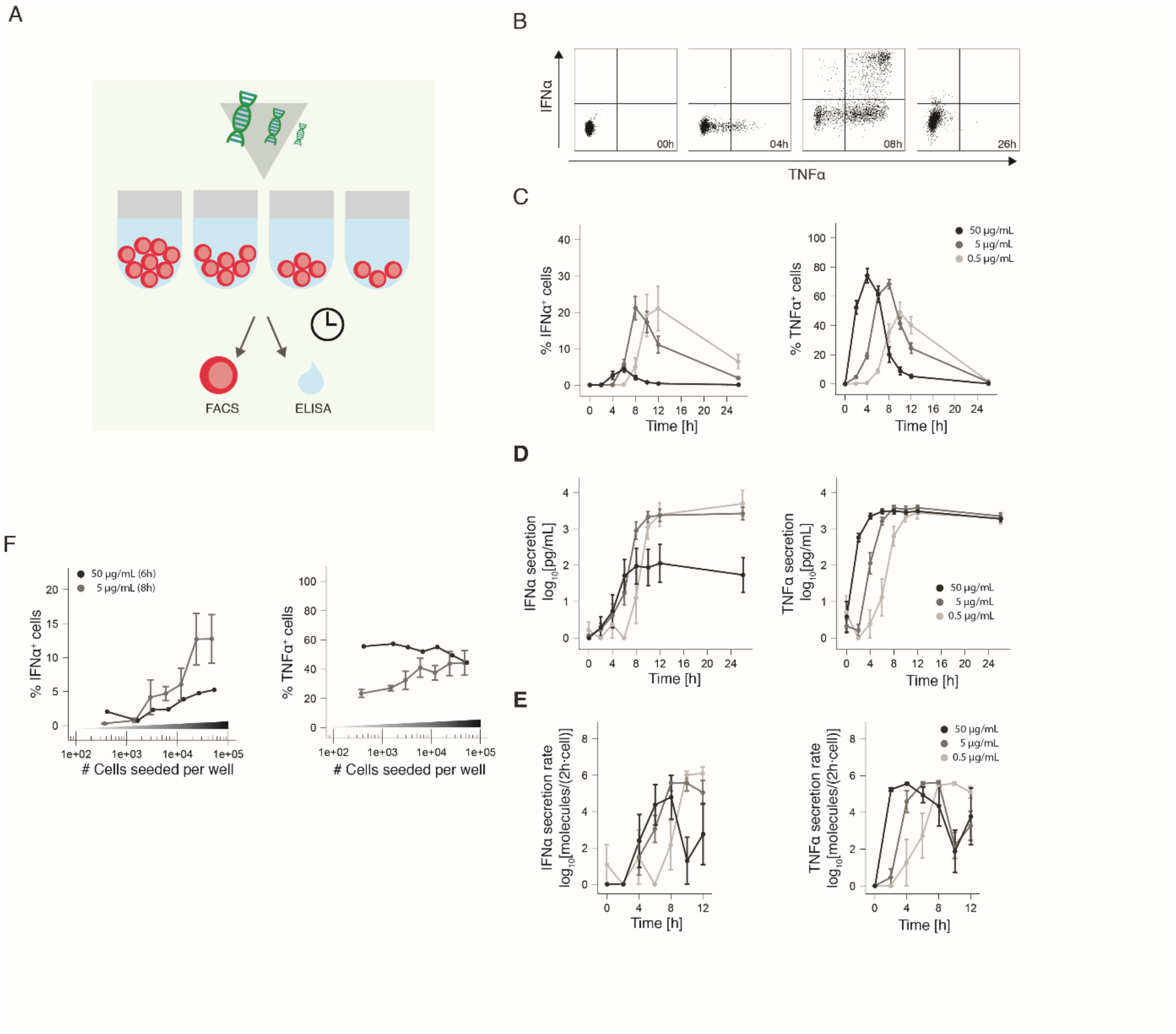

Supplementary Figure 9 – Kinetics of IFNα and TNFα secretion by pDCs in microtiter plate cultures. A) PDCs were stimulated with CpG-C in microtiter plates at varying cell densities or varying CpG-C concentrations. After incubation, cells were fixed, permeabilized, and stained for viability and cytokine expression. B) IFNα- and TNFα-expressing cells were detected using flow cytometry. C) PDCs were stimulated at a density of 25.000 cells/well. Shown is the fraction of cytokine-expressing pDCs plotted against incubation time. n>=5. At 50 µg/mL CpG-C less cells produce IFNα. Previous studies showed that an initial lag-phase before the onset of IFNα secretion is crucial to prime pDCs via autocrine or paracrine mechanisms.[2] The early onset of IFNα production by pDCs stimulated with 50 µg/mL CpG-C most-likely undercuts this threshold. In accordance with this, we observed robust IFNα responses when pre-treating pDCs for 2h with 500U/mL of IFNβ (Supplementary Figure 10). D) Supernatant was analyzed using ELISA. Shown is the concentration of IFNα and TNFα plotted against the incubation time. n>=5. E) Cytokine concentration from D) was combined with the number of cytokine-expressing cells, as determined via flow cytometric analysis in duplicate cultures (C), to calculate the average secretion rate of a single cell. Shown is the number of molecules, added to the

supernatant every two hours by a single cell, plotted against the incubation time. n>=5 F) PDCs were stimulated at different cell density and cytokine-expressing cells were detected using flow cytometry. n[5 µg/mL] = 3, n[50 µg/mL] = 1). B - F) Dots indicate mean, error bars indicate SEM.

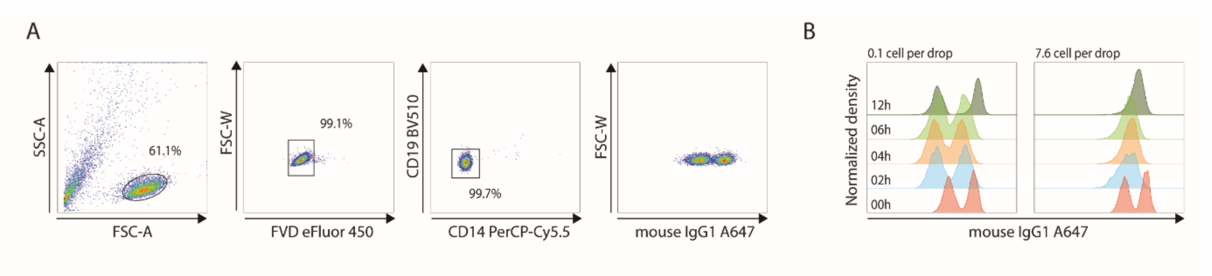

Supplementary Figure 10 – Cytokine capture-reagents can be exchanged between two cells encapsulated in the same droplet but not between two cells encapsulated in different droplets. PDCs were coated with capture reagent or left untreated and mixed at a 1:1 ratio. Subsequently, cells were encapsulated in picoliter droplets with either 0.1 or 7.6 cells per drop on average, and stimulated individually with 50 µg/mL CpG-C. A) Next to viability and surface marker expression, pDCs were also stained for cytokine capture reagent-coating using an antibody against mouse IgG1. B) Shown is the distribution of the fluorescence intensity of the capture reagent at each time point.

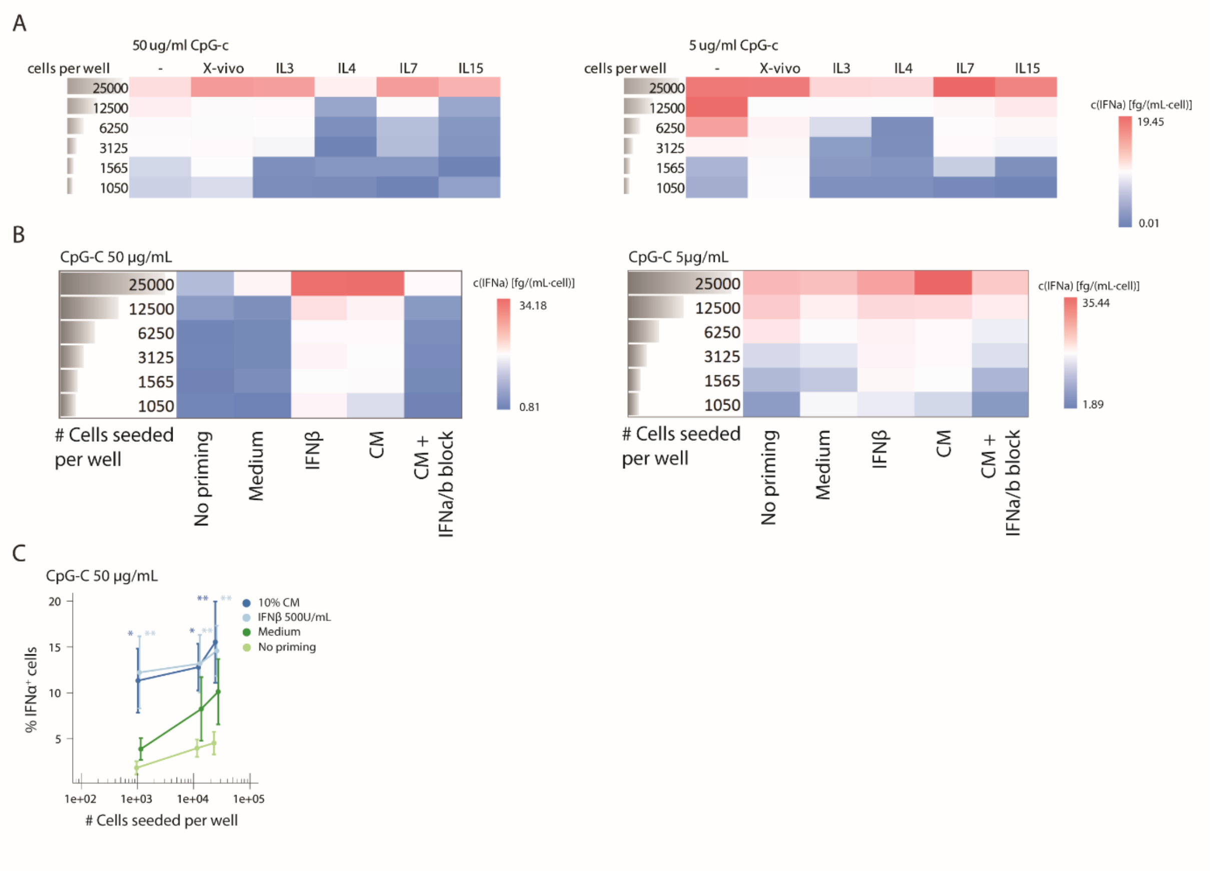

Supplementary Figure 11 – Effect of priming with different cytokines on IFNα production by pDCs. A, B) PDCs were incubated with fresh medium, conditioned medium or different cytokines (0.01 µg/ml IL-3, 60 µg/ml IL-4, 50 µg/ml IL-7, 20 µg/ml IL-15, 500 U/mL IFNβ) for two hours or left untreated. In some cases, cells were pre-incubated with blocking antibodies against IFNAR2 and CM was supplemented with neutralizing serum against IFNα and IFNβ (block). Subsequently, pDCs were stimulated with CpG-C in microtiter plates for 12h at varying cell densities, and cytokine concentration in supernatants was measured using ELISA. Shown is the log cytokine concentration relative to the number of seeded cells plotted against cell density and priming condition. A: n=1, B: n=3 C) PDCs were incubated with fresh medium, conditioned medium or 500 U/mL recombinant IFNβ for 2h or left untreated. Subsequently, pDCs were stimulated with CpG-C for 6h in microtiter plates at varying cell densities. After incubation, cells were fixed, permeabilized, and stained for viability and cytokine expression. IFNα- and TNFα-expressing cells were detected using flow cytometry. Shown is the fraction of IFNα-expressing cells plotted against the number of seeded cells. Values were compared to non-primed pDCs using the Mann-Whitney test. * $p < 0.05$, ** $p<0.01$. n>=6 Dots indicate mean, error bars indicate SEM.

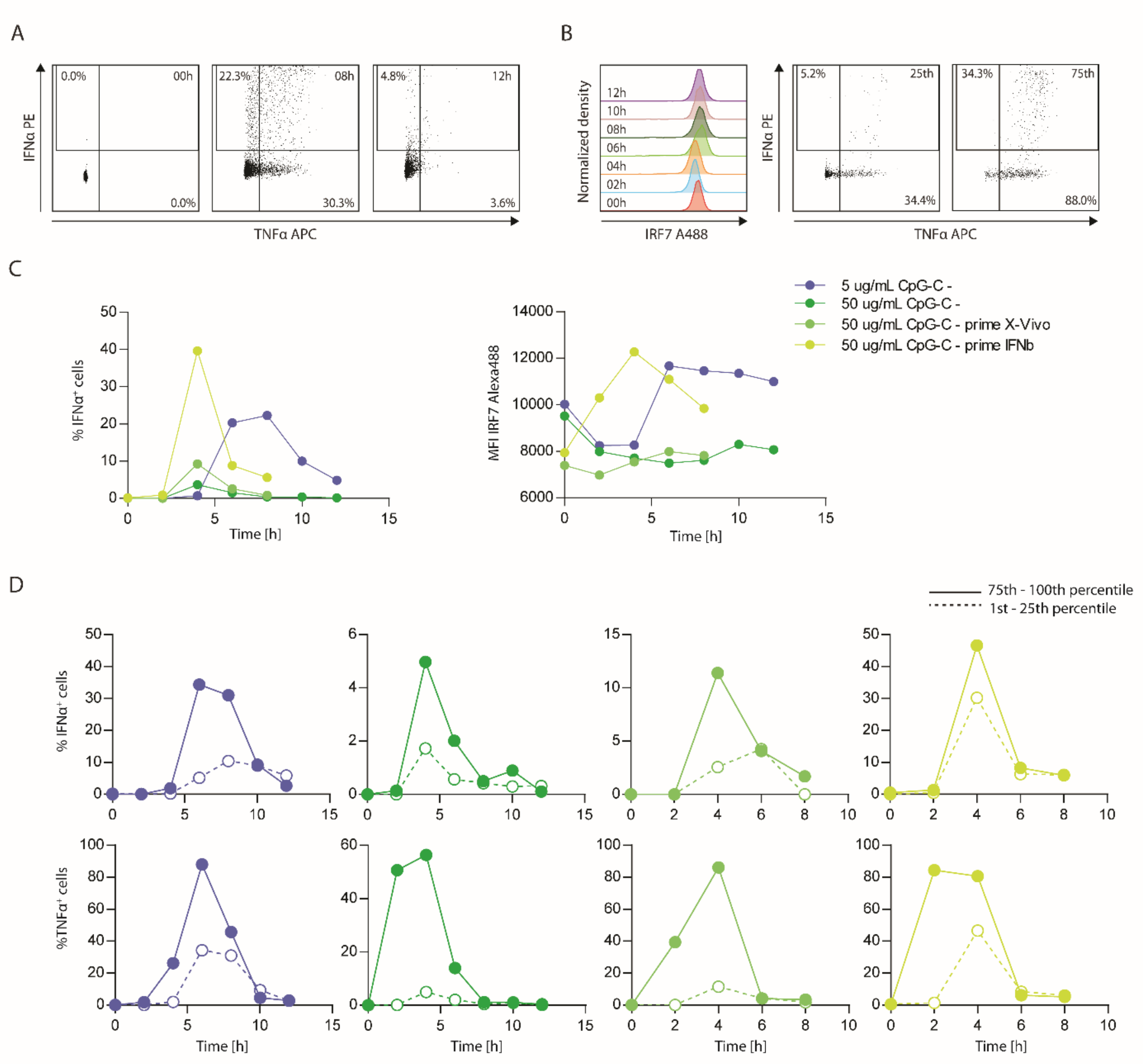

Supplementary Figure 12 – IRF7 expression dynamics in primed and stimulated pDCs. PDCs were incubated with fresh medium or 500 U/mL recombinant IFNβ for 2h or left untreated. Subsequently, pDCs were stimulated with CpG-C in microtiter plates at a density of 25.000. After incubation, cells were fixed, permeabilized, and stained for viability, cytokine expression and IRF7 expression. A, B) IFNα-, TNFα-, and IRF7-expressing cells were detected using flow cytometry. C) The fraction of cytokine producing cells or the fluorescence intensity of IRF7 was plotted against the incubation time. D) The 25% pDCs that had the lowest or highest expression of IRF7 were further selected and cytokine expression in those cells was analyzed separately. The fraction of cytokine producing cells for each group was plotted against the incubation time.

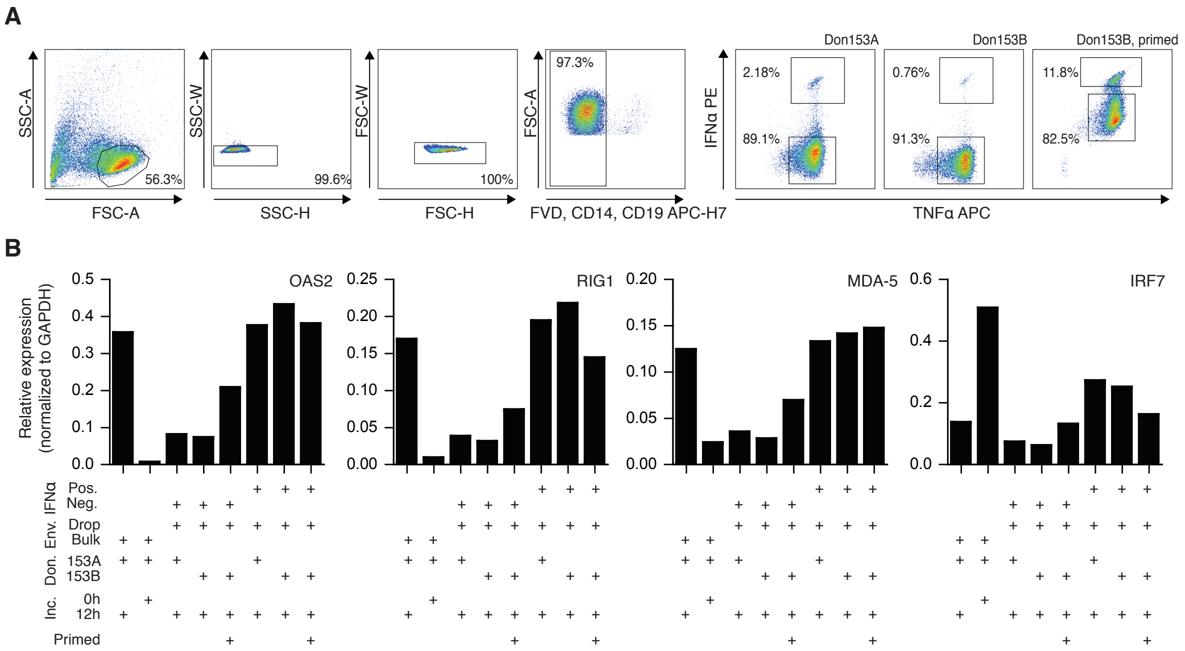

Supplementary Figure 13 – Expression of interferon stimulated genes in individually activated, sorted pDCs. PDCs were incubated with 40% conditioned medium for 2h or left untreated. Subsequently, cells were coated with capture reagent, encapsulated in picoliter droplets, and stimulated individually with 50 µg/mL CpG-C for 12h. Control cells were stimulated with 5 µg/mL CpG-C for 12h in a microtiter plate at a density of 25.000 cells (bulk) or left at 4°C (0h). A) After staining for viability and cytokine secretion, IFNα^{+} and IFNα^{-} cells were isolated using fluorescence activated cell sorting. Sorted cells were lysed, RNA was isolated, and the expression of the interferon stimulated genes OAS2, RIG1, MDA-5, and IRF7 as well as the house keeping gene GAPDH was determined via quantitative PCR. B) Shown are the expression levels relative to GAPDH plotted against treatment conditions.

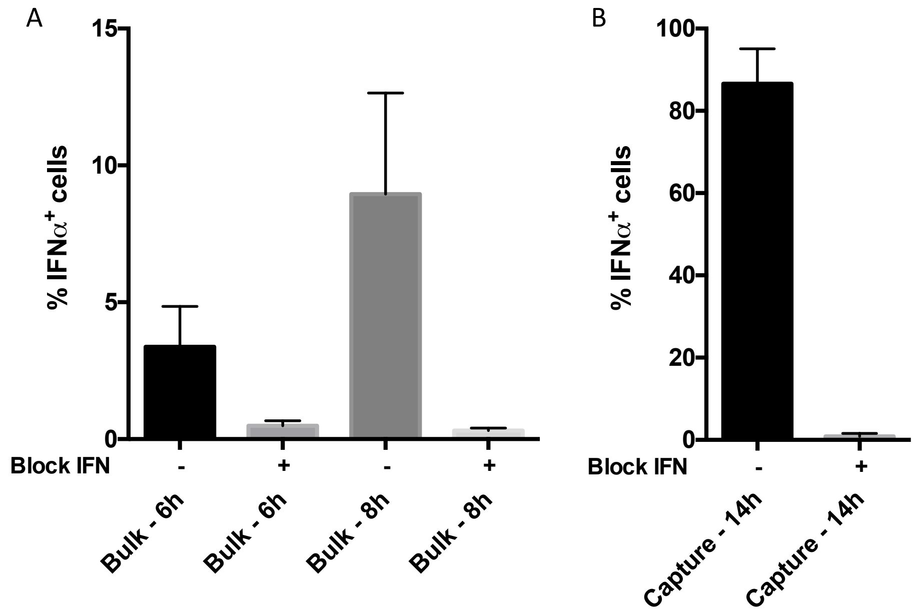

Supplementary Figure 14 – Effect of blocking paracrine type I IFN signaling on IFNα production by bulk cultured pDCs. A) PDCs were incubated with fresh medium (- block) or pre-incubated with blocking antibodies against IFNAR2 and the medium was supplemented with neutralizing serum against IFNα and IFNβ (+ block). Subsequently, pDCs were stimulated with CpG-C in microtiter plates for 6h or 8h at a density of 25.000 cells/well. After incubation, cells were fixed, permeabilized, and stained for viability and cytokine expression. IFNα-expressing cells were detected using flow cytometry. n=5 B) PDCs were coated with capture reagent, were pre-incubated with blocking antibodies against IFNAR2 and medium was supplemented with neutralizing serum against IFNα and IFNβ (block) prior to activation with 5 μg/mL CpG-C in bulk (25.000 cells/well) for 14h. IFNα-secreting cells were detected via flow cytometry. Shown is the fraction of IFNα-secreting cells plotted against treatment condition. n=5 A – B) Bars indicate mean, error bars indicate SEM.

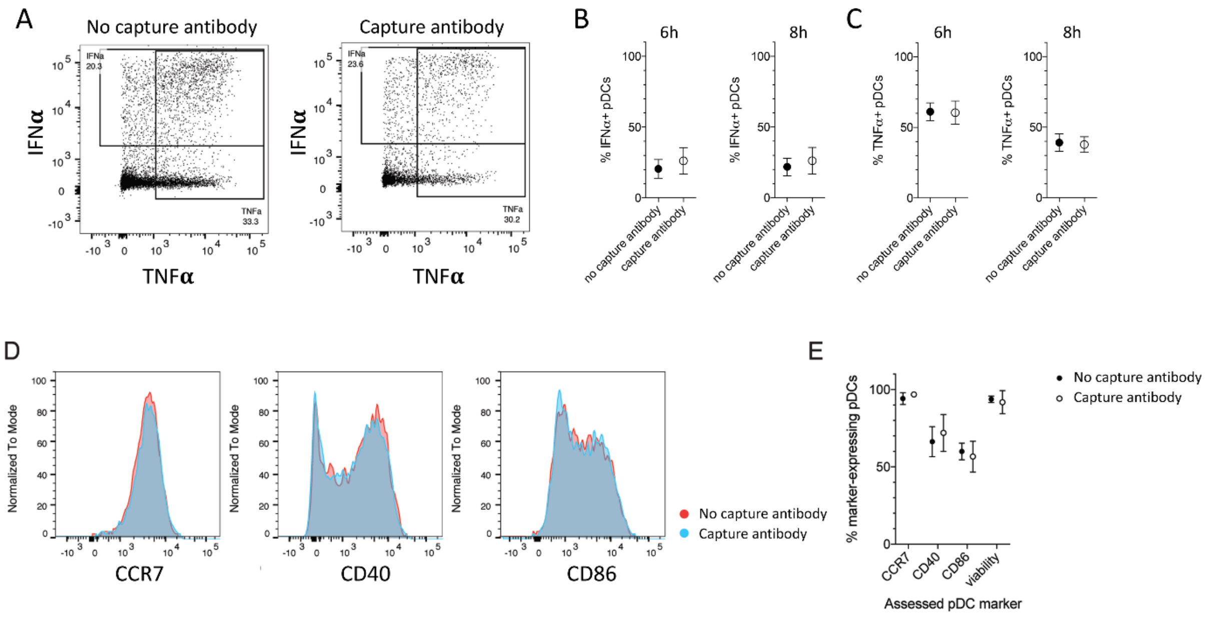

Supplementary Figure 15 – Effect of Cytokine Catch Reagents on cellular function and viability of bulk cultured pDCs. PDCs were coated with capture reagent or left untreated and subsequently activated with 5 µg/mL CpG-C in microtiter plates for 6h, 8h or 12h at a density of 25.000 cells/well. A) IFNα- and TNFα-secreting cells were detected via intracellular cytokine staining and flow cytometry after 8 hours and the result of 1 representative donor is shown. B, C) Shown is the fraction of IFNα- or TNFα-secreting cells plotted against treatment condition and stimulation for either 6 hours or 8 hours. Circles indicate mean, error bars indicate SEM, n=5. D) The expression of CCR7, CD40 and CD86 by differently treated pDCs was assessed after 12 hours of activation using flow cytometry. One representative experiment is shown. E) Shown is the viability and the expression of CCR7, CD40 and CD86 plotted against treatment condition. Circles indicate mean, error bars indicate SD, n=3.

Supplementary Methods

Supplementary Table 1 – Employed stimuli and cytokines

Stimulus	Comment	Standard conc bulk [µg/ml]	Standard conc drop [µg/ml]	Manufacturer
R848	Resiquimod	4	4	Enzo
CpG-A	ODN 2216	5	50	Enzo
CpG-B	ODN 2006	5	50	Enzo
CpG-C	ODN M362	5	50	Enzo
PMA	Phorbol 12-myristate 13-acetate		0.05	Calbiochem
Iono	Ionomycine		1	Sigma
IL-3	Recombinant human interleukin-3	0.1	0.1	Cellgenix
IFNβ	Recombinant human IFN-β	500 U/mL		Peprotech
IL-4	Human IL-4, premium grade	60		Miltenyi Biotec
IL-7		50		R&D
IL-15		20		Biolegend

Supplementary Table 2 – Employed primers

Gene	Name	Direction	Sequence (5' to 3')
GAPDH	hGAPDH FW	Forward	GAAGGTGAAGGTCGGAGT
GAPDH	hGAPDH RV	Reverse	AGATGGTGATGGGATTTC
IRF7	IRF-7.forw	Forward	GAGCCGTACCTGTCACCCT
IRF7	IRF-7.rev	Reverse	GGGCCGTATAGGAACGTGC
MDA5	MDA-5.forw	Forward	CAACATGGGCAGTGATTCAGG
MDA5	MDA-5.rev	Reverse	TGGGCAACTTCCATTTGGTAAG
OAS2	OAS2_fwd_1	Forward	AAGCCCTACGAAGAATGT
OAS2	OAS2_rev_1	Reverse	TTGGCTTCTCTTCTGATCCTGG
RIG-I	RIG-I.forw	Forward	TGTGCTCCTACAGGTTGTGGA
RIG-I	RIG-I.rev	Reverse	CACTGGGATCTGATTCGCAAAA

Supplementary Table 3 – Employed antibodies and cytokine detection kits

Antigen	Clone	Label	Dilution	Manufacturer
CD303	AC144	APC	1:10	Miltenyi Biotec
Lineage Cocktail 1		FITC	1:10	BD
CD3	SK7			
CD14	MϕP9			
CD16	3G8			
CD19	SJ25C1			
CD20	L27			
CD56	NCAM16.2			
CD304	AD5-17F6	PE	0.5 µL per 1M cells	Miltenyi Biotec
CD304	12C2	BV510	2 µL per 1M cells	Biolegend
HLA-DR	AC122	VioBlue, PE-Cy7	0.5 µL / 0.3 µL per 1M cells	Miltenyi Biotec
PD-L1	MIH1	BV421	1/40	BD
CD80	2D10.4	PerCp-eFluor710	1/20	eBioscience
CD86	2331	BV510	1/20	BD
CD40	5C3	PE-Cy7	1/90	BD
CCR7	150503	FITC	1/10	R&D
CD14	MϕP9	APC-H7	1/75	BD
CD14	M5E2	PerCP-Cy5.5	1/100	eBioscience
CD19	SJ25C1	APC-H7	1/20	BD
CD19	SJ25C1	BV510	1/50	BD
IFNα	LT27:295	PE	1/30	Miltenyi Biotec
TNFα	cA2	APC	1/30	Miltenyi Biotec
IRF7	12G9A36	Alexa488	1/25	Biolegend
Axl	108724	Alexa488	1/30	R&D
Siglec6	767329	APC	1/10	R&D
Mouse IgG1	Polyclonal	Alexa647	1/400	Life Technologies
IL-2 Cytokine capture reagent	Not disclosed	PE	1/10	Miltenyi Biotec
IFNγ Cytokine capture reagent	Not disclosed	FITC	1/10	Miltenyi Biotec
IFNα Cytokine capture reagent	Not disclosed	PE	1/10	Miltenyi Biotec
TNFα Cytokine capture reagent	Not disclosed	APC	1/10	Miltenyi Biotec
INFAR2	MMHAR-2		10 µg/mL	PBL Assay Science
IFNβ	Sheep serum		1000 NU/mL	PBL Assay Science
IFNα	Sheep serum		1000 NU/mL	PBL Assay Science

Supplementary Table 4 – Flow rates and droplet sizes

V (droplet)	Channel height	Flow rate continuous phase	Flow rate stimuli	Flow rate cells
41 pL	25 µm	1200 µL/h	150 µL/h	150 µL/h
75 pL	25 µm	900 µL/h	200 µL/h	200 µL/h
243 pL	50 µm	900 µL/h	200 µL/h	200 µL/h
1022 pL	80 µm	1000 µL/h	1000 µL/h	1000 µL/h
3121 pL	80 µm	150 µL/h	225 µL/h	225 µL/h

Supplementary code

Supplementary code 1 – RACE-ID2 Analysis for Figure 3

```
##########################################################################################################
################### Generation of the figure for scRNA analysis of stimulated pDCs
####################
##########################################################################################################
# Florian Wimmers; flowimmers@gmail.com; 22 April 2018


#### Requires the following packages:
# tsne
# pheatmap
# MASS
# cluster
# mclust
# flexmix
# lattice
# fpc
# RColorBrewer
# permute
# ampa
# locfit
# vegan


# run script "home made functions.R"
## load class definition and functions
source("./scripts/genRaceidObjct/RaceID_class.R")


# determine gene signatures for DC subsets according to Villani, Science, 2017
pdc <- c("NRP1__chr10", "CLEC4C__chr12", "GZMB__chr14", "SERPINF1__chr17", "ITM2C__chr2");
asdc <- c("AXL__chr19", "SIGLEC6__chr19", "PPP1R14A__chr19", "CD22__chr19", "DAB2__chr5");
cd1cA <- c("CD1C__chr1", "FCER1A__chr1", "CLEC10A__chr17", "ADAM8__chr10", "CD1D__chr1");
cd1cB <- c("S100A9__chr1", "S100A8__chr1", "VCAN__chr5", "LYZ__chr12", "ANXA1__chr9");
cd141 <- c("CLEC9A__chr12", "HLA-DPA1__chr6", "CADM1__chr11", "CAMK2D__chr4"); # not found:
C1ORF54
dc4 <- c("FCGR3A__chr1", "FTL__chr19", "SERPINA1__chr14", "LST1__chr6", "AIF1__chr6");



##############################################################
##### Load files and merge them into one data frame
##############################################################

# Here you load in the files, and the objects I give the name (FW14 etc.)
FW14<- read.csv("./data/scRNA data/raw/FW14_AHWTVGBGX2_S1_R2.TranscriptCounts.tsv",
row.names=1, sep="")
FW15<- read.csv("./data/scRNA data/raw/FW15_AHWTVGBGX2_S2_R2.TranscriptCounts.tsv",
row.names=1, sep="")
FW16<- read.csv("./data/scRNA data/raw/FW16_AHWTVGBGX2_S3_R2.TranscriptCounts.tsv",
row.names=1, sep="")

#Rename cols with correct plate coordinates
colnames(FW14)<- paste("FW14_", platenumbers_384, sep="")
colnames(FW15)<- paste("FW15_", platenumbers_384, sep="")
colnames(FW16)<- paste("FW16_", platenumbers_384, sep="")

#Merge datasets
prdata<- intersectmatrix(FW14,
                intersectmatrix(FW15,FW16))
```

```
#removal of noisy genes and the mitochondrial genes (chrM) from the genelist in the dataset, as well
as the spike-ins
prdata<- prdata[-grep("ERCC|chrM|MALAT1|KCNQ1OT1", rownames(prdata)) , ]



############################################################
##### Filter data
############################################################

# initialize SCseq object with transcript counts
sc <- SCseq(prdata)

# gene and cell count info before any filtering
dim(sc@fdata) # total genes and total cells
round(mean(colSums(sc@fdata))) #average no of transcripts per cell
summary(colSums(sc@fdata))
round(mean(colSums(sc@fdata != 0))) #average no of genes per cell
summary(colSums(sc@fdata != 0))

################# Initial filtering, clustering, outlier detection and removal of non-pDC DCs
# filtering of expression data
sc <- filterdata(sc, mintotal=1700, minexpr=1, minnumber=2, maxexpr=500, downsample=TRUE,
dsn=1, rseed=19000);

# gene and cell count info after filtering of genes expressed in only one cell, after
# removal of cells with less than 1700 transcripts, and after downsampling
dim(sc@fdata) # total genes and total cells
tmpG <- sc@fdata # temporary variable to revert cells to real 0
tmpG <- tmpG - 0.1
round(mean(colSums(tmpG))) #average no of transcripts per cell
summary(colSums(tmpG))
round(mean(colSums(sc@fdata != 0.1))) #average no of genes per cell
summary(colSums(sc@fdata != 0.1))


# kmedoids clustering
sc <- clustexp(sc, clustnr=20, bootnr=50, metric="pearson", do.gap=FALSE, sat=TRUE,
SE.method="Tibs2001SEmax", SE.factor=.25, B.gap=50, cln=0, rseed=17000,
FUNcluster="kmedoids")

# tsne
sc <- comptsne(sc,rseed=15555)

# raceID
sc <- findoutliers(sc, outminc=15,outlg=2,probthr=1e-5,thr=2**-(1:40),outdistquant=.95)

# inspection of clustering and raceID results
plottsne(sc,final=TRUE) # highlight final clusters in t-SNE map
plotexptsne(sc, dc4, n="CD141-CD1C- signature")
plotexptsne(sc, cd141, n="CD141 signature")
plotexptsne(sc, asdc, n="AS DC signature")
plotexptsne(sc, cd1cB, n="CD1C_B signature")
plotexptsne(sc, cd1cA, n="CD1C_A signature")
plotexptsne(sc, pdc, n="pDC signature")

# removal of non-pDC cluster from data set
nonPdc_indexnames <- names(sc@cpart[which(sc@cpart %in% 3)]) # get the index of all cells of a
certain cluster
write.csv(nonPdc_indexnames, "./data/scRNA data/temp/nonPdc-indexnames.csv"); # save those
indexes in a file
prdata <- prdata[ , !names(prdata) %in% nonPdc_indexnames ]; # remove the cells with those
indexes from data
sc <- SCseq(prdata)


################# Second filtering, clustering, outlier detection and removal of clean dataset
# filtering of expression data
```

```
sc <- filterdata(sc, mintotal=1700, minexpr=1, minnumber=2, maxexpr=500, downsample=TRUE,
dsn=1, rseed=19000);

# gene and cell count info after removing non-pDCs, after filtering of genes expressed in only one cell,
after
# removal of cells with less than 1700 transcripts, and after downsampling
dim(sc@fdata)
tmpG <- sc@fdata # temporary variable to revert cells to real 0
tmpG <- tmpG - 0.1
round(mean(colSums(tmpG))) #average no of transcripts per cell
summary(colSums(tmpG))
round(mean(colSums(sc@fdata != 0.1))) #average no of genes per cell
summary(colSums(sc@fdata != 0.1))

# kmedoids clustering
sc <- clustexp(sc, clustnr=20, bootnr=50, metric="pearson", do.gap=FALSE, sat=TRUE,
SE.method="Tibs2001SEmax", SE.factor=.25, B.gap=50, cln=0, rseed=17000,
FUNcluster="kmedoids")

# tsne
sc <- comptsne(sc,rseed=15555)

# raceID
sc <- findoutliers(sc, outminc=15,outlg=2,probthr=1e-5,thr=2**-(1:40),outdistquant=.95)



################################################################
##### Quality control plots
################################################################

########### Clustering
# plot within-cluster dispersion as a function of the cluster number: only if sat == TRUE
plotsaturation(sc,disp=TRUE)
# plot change of the within-cluster dispersion as a function of the cluster number: only if sat == TRUE
plotsaturation(sc)
# Jaccard's similarity of k-medoids clusters
plotjaccard(sc)
# Identiy heatmap
clustheatmap(sc,final=FALSE,hmethod="single")
# tsne plot
plottsne(sc,final=FALSE) # highlight k-medoids clusters in t-SNE map

########### Outlier
# barchart of outlier probabilities
plotoutlierprobs(sc)
# regression of background model
plotbackground(sc)
# dependence of outlier number on probability threshold (probthr)
plotsensitivity(sc)



################################################################
##### Detection of CD2 subset
################################################################
hlgene_log("LYZ__chr12") # enriched in CD2hiCD5+CD81+ from 2017 PNAS paper and from 2008 JI
hlgene_log("ANXA1__chr9") # enriched in CD2hiCD5+CD81+ from 2017 PNAS paper
hlgene_log("COTL1__chr16") # enriched in CD2hiCD5+CD81+ from 2017 PNAS paper
hlgene_log("CD86__chr3") # expressed by CD2hiCD5+ in steady state from 2013 PLOS One
```

Supplementary code 2 - Diff Gene expression for Figure 4

```
#############################################################################
###################################
################### Compare gene expression between different clusters using deSEQ
####################
#############################################################################
###################################
# Florian Wimmers; flowimmers@gmail.com; 22 April 2018; adapted from Muraro et al, Cell
Systems, 2016

source("./scripts/diffGeneExp/functions.R")


#### differential gene expression analysis with diffexpnb ####
x <- c(2,3,4,5,6,7,8) # clusters to loop through

for (i in 1:length(x)) {

  ## (2) compare two or more clusters with one another
  cluster1<-c(1) # pick cluster(s) in 1st group
  cluster2<-c(x[i]) # pick cluster(s) in 2nd group
  name1<-paste("resting pDC - cl",paste(cluster1, collapse = " ", sep="")) # will generate a name for
plotting and writing files
  name2<-paste("diverging - cl",paste(cluster2,collapse= " ", sep=""))
  a<-diffexpnb(sc@fdata,names(sc@fdata)[sc@cpart %in% cluster1],names(sc@fdata)[sc@cpart
%in% cluster2],norm=FALSE,logrec=FALSE,
          vfit=sc@background$vfit,method="pooled")

  # following 1 or 2, plot the results, order them in a ranked list (either by expression or p val) and
write to file
  pval<- 10**-8 # choose padjusted value cutoff

  date <- format(Sys.time(), "%y%m%d")
  pdf(file=paste("./temp/", "MICRO_volcano_",name1,"_vs_",name2,"_pval_",pval,".pdf", sep="")) #
open device to save plot
  plotdiffgenesnb(a, xname=name1, yname=name2, pthr = pval ,lthr=1, mthr=1, show_names=F,
padj=T) # plot diff genes
  dev.off()

  diffgen<-a$res[which(a$res$padj < pval),] #select significant genes
  # diffgen.filter<-diffgen[order(diffgen$padj,decreasing=F),] # order on padjusted value
  # diffgen.filter<-diffgen[order(diffgen$baseMean,decreasing=T),] # or order on mean expression
  diffgen.filter<-diffgen[order(diffgen$foldChange,decreasing=T),] # or order on fold change
expression

  diffgen.up<-subset(diffgen.filter,diffgen.filter$log2FoldChange > 1.5) # subset only upregulated
  cat(paste(nrow(diffgen.up), "genes are significant\n"))

  #write results to textfile
  rownames(diffgen.up)<-sapply(rownames(diffgen.up),chop_chr) # remove __chr part from
rownames
  write.table(diffgen.up,paste("./temp/", "MICRO_diffGenUp_",name1,"_vs_",name2,"_pval_",pval,".txt",
sep=""),sep="\t", col.names=NA) # write results



  diffgen.down<-subset(diffgen.filter,diffgen.filter$log2FoldChange < -1.5) # subset only
downregulated
  cat(paste(nrow(diffgen.down), "genes are significant\n"))

  #write results to textfile
  rownames(diffgen.down)<-sapply(rownames(diffgen.down),chop_chr) # remove __chr part from
rownames
  write.table(diffgen.down,paste("./temp/",
"MICRO_diffGenDown_",name1,"_vs_",name2,"_pval_",pval,".txt", sep=""),sep="\t", col.names=NA) #
write results
}
```

Supplementary code 3 - RACE-ID2 Analysis for Supplementary Figure 8

```
###################################################################################################
################### Generation of the figure for scRNA analysis of stimulated pDCs ####################
###################################################################################################
# Florian Wimmers; flowimmers@gmail.com; 22 April 2018

#### Requires the following packages:
# tsne
# pheatmap
# MASS
# cluster
# mclust
# flexmix
# lattice
# fpc
# RColorBrewer
# permute
# ampa
# locfit
# vegan


# run script "home made functions.R"
## load class definition and functions
source("./scripts/genRaceidObjct/RaceID_class.R")


# determine gene signatures for DC subsets according to Villani, Science, 2017
pdc <- c("NRP1__chr10", "CLEC4C__chr12", "GZMB__chr14", "SERPINF1__chr17", "ITM2C__chr2");
asdc <- c("AXL__chr19", "SIGLEC6__chr19", "PPP1R14A__chr19", "CD22__chr19", "DAB2__chr5");
cd1cA <- c("CD1C__chr1", "FCER1A__chr1", "CLEC10A__chr17", "ADAM8__chr10", "CD1D__chr1");
cd1cB <- c("S100A9__chr1", "S100A8__chr1", "VCAN__chr5", "LYZ__chr12", "ANXA1__chr9");
cd141 <- c("CLEC9A__chr12", "HLA-DPA1__chr6", "CADM1__chr11", "CAMK2D__chr4"); # not found: C1ORF54
dc4 <- c("FCGR3A__chr1", "FTL__chr19", "SERPINA1__chr14", "LST1__chr6", "AIF1__chr6");



#################################################################
##### Load files and merge them into one data frame
#################################################################

# Here you load in the files, and the objects I give the name (FW14 etc.)
FW07<- read.csv("./data/scRNA data/raw/Fw7_AHNGTJBGX2_S3_R2.TranscriptCounts.tsv", row.names=1, sep="")
FW09<- read.csv("./data/scRNA data/raw/Fw9_AHNGTJBGX2_S4_R2.TranscriptCounts.tsv", row.names=1, sep="")
FW12<- read.csv("./data/scRNA data/raw/Fw12_AHNGTJBGX2_S5_R2.TranscriptCounts.tsv", row.names=1, sep="")
FW13<- read.csv("./data/scRNA data/raw/Fw13_AHNGTJBGX2_S6_R2.TranscriptCounts.tsv", row.names=1, sep="")
FW14<- read.csv("./data/scRNA data/raw/FW14_AHWTVGBGX2_S1_R2.TranscriptCounts.tsv", row.names=1, sep="")
FW15<- read.csv("./data/scRNA data/raw/FW15_AHWTVGBGX2_S2_R2.TranscriptCounts.tsv", row.names=1, sep="")
FW16<- read.csv("./data/scRNA data/raw/FW16_AHWTVGBGX2_S3_R2.TranscriptCounts.tsv", row.names=1, sep="")

#Rename cols with correct plate coordinates
colnames(FW07)<- paste("FW07_", platenumbers_384, sep="")
colnames(FW09)<- paste("FW09_", platenumbers_384, sep="")
colnames(FW12)<- paste("FW12_", platenumbers_384, sep="")
colnames(FW13)<- paste("FW13_", platenumbers_384, sep="")
```

```
colnames(FW14)<- paste("FW14_", platenumbers_384, sep="")
colnames(FW15)<- paste("FW15_", platenumbers_384, sep="")
colnames(FW16)<- paste("FW16_", platenumbers_384, sep="")

#Merge datasets
prdata<- intersectmatrix(FW07,
              intersectmatrix(FW09,
                     intersectmatrix(FW12,
                            intersectmatrix(FW13,
                                   intersectmatrix(FW14,
                                          intersectmatrix(FW15,FW16))))))

#removal of noisy genes and the mitochondrial genes (chrM) from the genelist in the dataset, as well
as the spike-ins
prdata<- prdata[-grep("ERCC|chrM|MALAT1|KCNQ1OT1", rownames(prdata)) , ]



#############################################################
##### Filter data
#############################################################

# initialize SCseq object with transcript counts
sc <- SCseq(prdata)

# gene and cell count info before any filtering
dim(sc@fdata) # total genes and total cells
round(mean(colSums(sc@fdata))) #average no of transcripts per cell
summary(colSums(sc@fdata))
round(mean(colSums(sc@fdata != 0))) #average no of genes per cell
summary(colSums(sc@fdata != 0))

# removal of non-pDC cluster from data set
nonPdc_indexnames <- read.csv("./data/scRNA data/temp/nonPdc-indexnames.csv", row.names=1,
header = TRUE); # load indexes of excluded cells
prdata <- prdata[ , !names(prdata) %in% nonPdc_indexnames$x ]; # remove the cells with those
indexes from data
sc <- SCseq(prdata)

# gene and cell count info after removal of non-pDC cells from donor 174
dim(sc@fdata) # total genes and total cells
round(mean(colSums(sc@fdata))) #average no of transcripts per cell
summary(colSums(sc@fdata))
round(mean(colSums(sc@fdata != 0))) #average no of genes per cell
summary(colSums(sc@fdata != 0))

################# Filtering, clustering, outlier detection and removal of non-pDC DCs
# filtering of expression data
sc <- filterdata(sc, mintotal=1700, minexpr=1, minnumber=2, maxexpr=500, downsample=TRUE,
dsn=1, rseed=19000);

# gene and cell count info after filtering of genes expressed in only one cell, after
# removal of cells with less than 1700 transcripts, and after downsampling
dim(sc@fdata) # total genes and total cells
tmpG <- sc@fdata # temporary variable to revert cells to real 0
tmpG <- tmpG - 0.1
round(mean(colSums(tmpG))) #average no of transcripts per cell
summary(colSums(tmpG))
round(mean(colSums(sc@fdata != 0.1))) #average no of genes per cell
summary(colSums(sc@fdata != 0.1))

# kmedoids clustering
sc <- clustexp(sc, clustnr=20, bootnr=50, metric="pearson", do.gap=FALSE, sat=TRUE,
SE.method="Tibs2001SEmax", SE.factor=.25, B.gap=50, cln=0, rseed=17000,
FUNcluster="kmedoids")

# tsne
sc <- comptsne(sc,rseed=15555)

# raceID
```

```
sc <- findoutliers(sc, outminc=15,outlg=2,probthr=1e-5,thr=2**-(1:40),outdistquant=.95)

# inspection of clustering and raceID results
plottsne(sc,final=TRUE) # highlight final clusters in t-SNE map
plotexptsne(sc, dc4, n="CD141-CD1C- signature")
plotexptsne(sc, cd141, n="CD141 signature")
plotexptsne(sc, asdc, n="AS DC signature")
plotexptsne(sc, cd1cB, n="CD1C_B signature")
plotexptsne(sc, cd1cA, n="CD1C_A signature")
plotexptsne(sc, pdc, n="pDC signature")



#################################################################
##### Quality control plots
#################################################################

########### Clustering
# plot within-cluster dispersion as a function of the cluster number: only if sat == TRUE
plotsaturation(sc,disp=TRUE)
# plot change of the within-cluster dispersion as a function of the cluster number: only if sat == TRUE
plotsaturation(sc)
# Jaccard's similarity of k-medoids clusters
plotjaccard(sc)
# Identiy heatmap
clustheatmap(sc,final=FALSE,hmethod="single")
# tsne plot
plottsne(sc,final=FALSE) # highlight k-medoids clusters in t-SNE map

########### Outlier
# barchart of outlier probabilities
plotoutlierprobs(sc)
# regression of background model
plotbackground(sc)
# dependence of outlier number on probability threshold (probthr)
plotsensitivity(sc)



#################################################################
##### Detection of CD2 subset
#################################################################
hlgene_log("LYZ__chr12") # enriched in CD2hiCD5+CD81+ from 2017 PNAS paper and from 2008 JI
hlgene_log("ANXA1__chr9") # enriched in CD2hiCD5+CD81+ from 2017 PNAS paper
hlgene_log("COTL1__chr16") # enriched in CD2hiCD5+CD81+ from 2017 PNAS paper
hlgene_log("CD86__chr3") # expressed by CD2hiCD5+ in steady state from 2013 PLOS One
```

References

1. Villani, A.C. *et al.* Single-cell RNA-seq reveals new types of human blood dendritic cells, monocytes, and progenitors. *Science* **356** (2017).

2. Kim, S. *et al.* Self-priming determines high type I IFN production by plasmacytoid dendritic cells. *Eur J Immunol* **44**, 807-818 (2014).
