# Supplementary material for: Single-cell analysis reveals that stochasticity and paracrine signaling control interferon-alpha production by plasmacytoid dendritic cells
Source: Nat Commun. 2018 Aug 20;9:3317. doi: 10.1038/s41467-018-05784-3 (PMC6102223; doi:10.1038/s41467-018-05784-3)
Supplement: Supplementary file 3 — Description of Additional Supplementary Files [file 41467_2018_5784_MOESM3_ESM.pdf]

Description of Additional Supplementary Files

File Name: Supplementary Data 1
Description: Downregulated genes for Fig 4. In Figure 4, differentially regulated genes in cells from all activated clusters compared to cluster 1 were identified using DEseq. For each cluster, significantly downregulated genes are stored in this table.

File Name: Supplementary Data 2
Description: Upregulated genes for Fig 4. In Figure 4, differentially regulated genes in cells from all activated clusters compared to cluster 1 were identified using DEseq. For each cluster, significantly upregulated genes are stored in this table.

File Name: Supplementary Data 3
Description: Kegg Terms for Fig 4. In Figure 4, gene enrichment analyses were conducted by submitting, for each cluster, a list with up to 50 of the most upregulated genes to the DAVID 6.7 web platform. Significantly enriched Kegg pathway terms for each cluster are stored in this table.

File Name: Supplementary Data 4
Description: Go Terms for Fig 4. In Figure 4, gene enrichment analyses were conducted by submitting, for each cluster, a list with up to 50 of the most upregulated genes to the DAVID 6.7 web platform. Significantly enriched GO terms for each cluster are stored in this table
